# Physical Activity and Neighborhood Socioeconomic Disadvantage Among Low-Income African Americans

**DOI:** 10.1093/geroni/igaa057.1279

**Published:** 2020-12-16

**Authors:** Dextiny McCain, Adrienne Aiken Morgan, Karon Phillips, Keith Whitfield

**Affiliations:** 1 North Carolina A&T State University, Kernersville, North Carolina, United States; 2 North Carolina A&T State University, Greensboro, North Carolina, United States; 3 UMBC, Baltimore, Maryland, United States; 4 Wayne State University, Detroit, Michigan, United States

## Abstract

Research shows regular physical activity (PA) is associated with better health and longevity; however, few studies consider contextual factors related to PA among African American (AA) older adults living in socioeconomically disadvantaged neighborhoods. The Physical and Cognitive Health Pilot Study (n=50) was used to examine associations between PA and level of neighborhood socioeconomic disadvantage among sedentary, AA older adults from four public housing communities in Durham, NC and Annapolis, MD (mean age=64.5; SD=10.42; 72% women). Participants were administered the Community Healthy Activities Model Program for Seniors (CHAMPS), a self-report questionnaire measuring weekly frequency and duration of PAs. Neighborhood socioeconomic disadvantage was defined by the Neighborhood Atlas Area Deprivation Index (ADI), which ranks neighborhoods according to Census block group/neighborhoods within each state and nationally. For the present sample, two of the Durham housing facilities were located in communities in the most disadvantaged block groups. Meanwhile, one Durham location and the Annapolis community were located in the least disadvantaged block groups. Bivariate correlations showed greater neighborhood socioeconomic disadvantage was associated with less participation in various PAs (p<.05). Next, ANOVA revealed the Annapolis group participated in statistically significantly more PAs, including visiting the senior center, church attendance, and light gardening (p<.05) compared to the most disadvantaged groups. The present findings suggest there are benefits to living in advantaged contexts despite lower-income status. These findings also suggest barriers within disadvantaged neighborhoods that limit access to recreational activities favorable to health status. Future research should address ways to overcome such barriers.

